# Data set and machine learning models for the classification of network traffic originators

**DOI:** 10.1016/j.dib.2022.107968

**Published:** 2022-03-03

**Authors:** Daniele Canavese, Leonardo Regano, Cataldo Basile, Gabriele Ciravegna, Antonio Lioy

**Affiliations:** a Dipartimento di Automatica e Informatica, Politecnico di Torino, Torino 10129, Italy; b Dipartimento di Ingegneria dell'Informazione, Universita` degli Studi di Firenze, Siena 53100, Italy

**Keywords:** Network traffic anomaly, Intrusion detection, Machine learning, DoS attacks, Web crawling

## Abstract

The widespread adoption of encryption in computer network traffic is increasing the difficulty of analyzing such traffic for security purposes. The data set presented in this data article is composed of network statistics computed on captures of TCP flows, originated by executing various network stress and web crawling tools, along with statistics of benign web browsing traffic. Furthermore, this data article describes a set of Machine Learning models, trained using the described data set, which can classify network traffic by the tool category (network stress tool, web crawler, web browser), the specific tool (e.g., Firefox), and also the tool version (e.g., Firefox 68) used to generate it. These models are compatible with the analysis of traffic with encrypted payload since statistics are evaluated only on the TCP headers of the packets. The data presented in this article can be useful to train and assess the performance of new Machine Learning models for tool classification.

## Specifications Table

**Table:** 

Subject	Cryptography and Cybersecurity
Specific subject area	Cyber-threat and Anomaly Detection
Type of data	Table, Chart
How data were acquired	Data was gathered by performing real live captures by our research group
Data format	Analyzed, Filtered
Parameters for data collection	All tools used to generate the dataset have been used with default parameters.
Description of data collection	(1) Traffic generated by a set of scripts launching various network stress and web crawling tools, along with traffic obtained through manual web browsing of various users, have been captured using tshark. 2) A set of network statistics on the resulting traffic captures evaluated using the tstat tool. The client/server IPs and TCP ports have been removed from the data set, both for anonymization purposes and because they are not useful to train the model.
Data source location	City: Turin, Cuneo; Country: Italy
Data accessibility	Repository name: Encryption agnostic classifiers of traffic originators and their application to anomaly detection - data sets and models Data identification number: 10.5281/zenodo.5797882 Direct URL to data: 10.5281/zenodo.5797882
Related research article	D. Canavese, L. Regano, C. Basile, G. Ciravegna, A. Lioy, Encryption agnostic classifiers of traffic originators and their application to anomaly detection, Computers and Electrical Engineering, 10.1016/j.compeleceng.2021.107621

## Value of the Data

• This data may be used as a benchmark for developing Machine Learning models aimed at obtaining information about the tools that originated sniffed network traffic. Presently, no benchmark data are available for researchers wanting to perform this type of classification. These models are of interest for developers of security monitoring systems, like Intrusion Detection Systems. Several types of attacks, e.g., Distributed Denial of Service and web crawling attacks, are launched using *ad hoc* tools. Therefore, getting information about the tools that originate the traffic can improve the detection abilities of these monitoring systems.
• These data are valuable as a data set for researchers interested in training Machine Learning models designed to obtain information about the tools that originate the sniffed traffic. Moreover, these data may serve for hyperparameters’ optimization processes.
• Since several of the trained Machine Learning models are based on neural networks, these data also may be used to speed up the training of new neural networks via transfer learning.
• These data allow assessing the results of research presented in [1], which first aimed at obtaining information about the tools that originated sniffed network traffic.

## 1 Data Description

### 1.1 Data set

This section reports several statistics about the data set Table 1. lists the tools used to generate the traffic considered in the presented data set Table 2. reports the features that have been used to train and test the Machine Learning models.

**Table 1:** Tools used to generate the traffic considered in the experiments.

application	category	Windows	Linux
Chrome 48	browser	☑	☑
Chrome 68	browser	☑	☑
Firefox 42	browser	☑	☑
Firefox 62	browser	☑	☑
Firefox 68	browser	☒	☑
Edge 42	browser	☑	☒
Opera 62	browser	☑	☒

GoldenEye 3.49.2	stress tool	☑	☑
HULK 1.0	stress tool	☑	☑
RudyJS 1.0.0	stress tool	☑	☑
SlowHTTPTest 1.6	stress tool	☒	☑
SlowLoris 7.70	stress tool	☑	☑

Curl 7.55	web crawler	☑	☑
GrabSite 2.1.16	web crawler	☒	☑
Httrack 3.49.2	web crawler	☑	☑
Wget 1.19	web crawler	☑	☑
Wpull 2.0.1	web crawler	☑	☑

**Table 2:** TCP statistics used as classification features.

feature		unit
1	# packets (both directions)	*packets*
2	# packets with payload (both directions)	*packets*
3	# retransmitted packets (both directions)	*packets*
4	# out of sequence packets (both directions)	*packets*
5	# packets with ACK set (both directions)	*packets*
6	# packets with ACK set and no payload (both directions)	*packets*
7	# packets with FIN set (both directions)	*packets*
8	# packets with RST set (both directions)^1^	*packets*
9	# packets with SYN set (both directions)	*packets*
10	# payload bytes excluding retransmissions (both directions)	*bytes*
11	# payload bytes including retransmissions (both directions)	*bytes*
12	# retransmitted bytes (both directions)	*bytes*
13	flow duration	*ms*
14	relative time of first payload packet (both directions)	*ms*
15	relative time of last payload packet (both directions)	*ms*
16	relative time of first ACK packet (both directions)	*ms*
17	TCP connection correctly terminated	*boolean*

1 This can be only 0 or 1 since a proper TCP implementation will reset a connection after receiving an RST packet.

Finally, several statistics, grouped by labels, are reported: the average number of packets and bytes sent by the client or server and the average connection duration in milliseconds Tables 3. and 4, respectively, report the averages for all the tools and their instances in the data set.

**Table 3:** Means of some features for the tool in our data set.

	sent by client		sent by server		
tool	packets	bytes	packets	bytes	duration [*ms*]
chrome	30.605	2587.957	43.548	46,727.950	36,023.258
curl	48.260	539.707	71.030	91280.696	608.296
edge	23.135	2024.088	21.693	21782.879	12693.361
firefox	40.620	2744.452	60.108	71260.752	24748.461
goldeneye	13.061	800.137	21.071	24220.021	1409.918
grabsite	368.901	3929.413	583.037	1803017.128	15427.538
httrack	16.424	1009.946	21.192	23018.443	2517.901
hulk	5.711	573.383	4.576	2659.303	5909.654
opera	25.263	1914.553	47.881	53419.715	40428.032
rudy	11.332	713.800	10.997	3342.403	15770.126
slowhttptest	8.640	1406.865	6.826	3494.015	11974.112
slowloris	5.280	164.620	3.890	47.859	13641.368
wget	129.312	2134.542	246.652	328985.405	2756.862
wpull	115.476	1239.060	214.299	296092.743	8558.179

**Table 4:** Means of some features for the tool instance in our data set.

	sent by client		sent by server		
tool instance	packets	bytes	packets	bytes	duration [*ms*]
chrome-48.0.2564.109	31.145	2337.780	41.950	41918.843	34469.858
chrome-68.0.3440.84	29.843	2941.171	45.803	53517.726	38216.437
curl-7.55.1	31.203	649.631	53.382	65814.020	431.330
curl-7.61.0	67.340	416.752	90.771	119766.433	806.241
edge-42.17134.1.0	23.135	2024.088	21.693	21782.879	12693.361
firefox-42.0	37.651	3162.645	57.196	66622.033	24610.871
firefox-62.0	49.066	3359.130	72.157	84900.460	33731.822
firefox-68.0	30.374	1155.053	43.732	54509.414	9834.131
goldeneye-2.1	13.061	800.137	21.071	24220.021	1409.918
grabsite-2.1.16	368.901	3929.413	583.037	1803017.128	15427.538
httrack-3.49.2	16.424	1009.946	21.192	23018.443	2517.901
hulk-1.0	5.711	573.383	4.576	2659.303	5909.654
opera-62.0.3331.66	25.263	1914.553	47.881	53419.715	40428.032
rudy-1.0.0	11.332	713.800	10.997	3342.403	15770.126
slowhttptest-1.6	8.640	1406.865	6.826	3494.015	11974.112
slowloris-0.1.4	5.404	164.220	3.973	48.299	13392.927
slowloris-0.1.5	5.159	165.008	3.810	47.434	13881.584
wget-1.11.4	92.605	1024.641	184.089	249312.020	2076.603
wget-1.19.5	176.123	3549.987	326.437	430592.154	3624.391
wpull-2.0.1	115.476	1239.060	214.299	296092.743	8558.179

### 1.2 Classifiers

This section reports several statistics and plots about the models for classifying the traffic into various classes. Three different models have been considered for each classification task: a random forest (via the RandomForestClassifier class in scikit-learn), an extra-trees (via the ExtraTreesClassifier class in scikit-learn), and a neural network (a custom class implemented in PyTorch and skorch). The optimization process was performed using the hyperopt package using a Bayesian optimization procedure.

For each classifier, the following data are reported:

• The plots showing the values of the *R_k_* statistics as our Bayesian hyper-parameters optimization process progressed (Fig. 1, Fig. 2, Fig. 3, Fig. 4, Fig. 5, Fig. 6, Fig. 7, Fig. 8, Fig. 9).

**Fig. 1:**
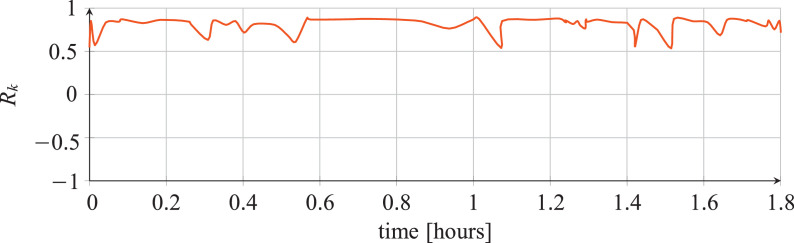
Hyper-parameters optimization plot for the category classifier based on random forest.

**Fig. 2:**
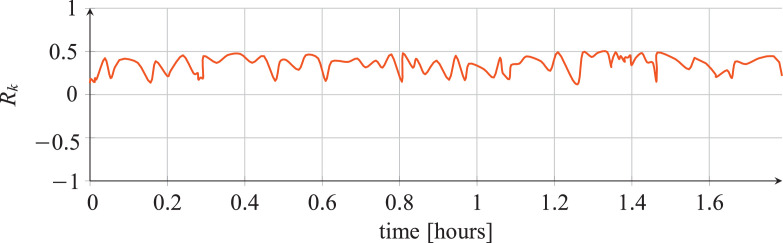
Hyper-parameters optimization plot for the category classifier based on extra-trees.

**Fig. 3:**
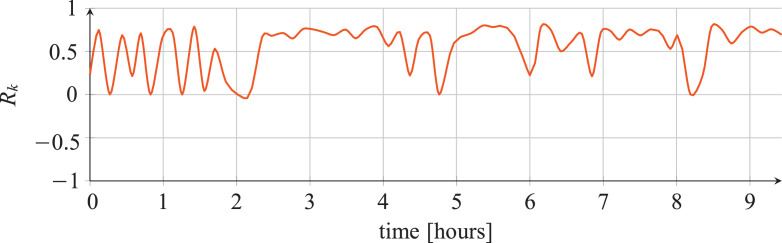
Hyper-parameters optimization plot for the category classifier based on neural network.

**Fig. 4:**
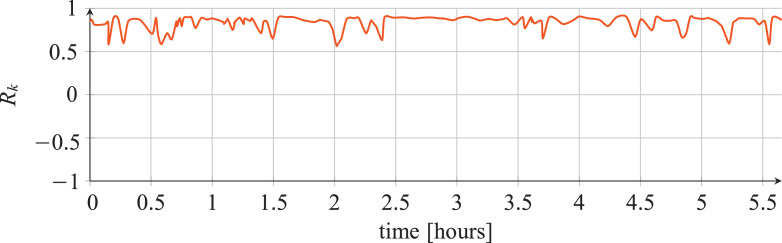
Hyper-parameters optimization plot for the tool classifier based on random forest.

**Fig. 5:**
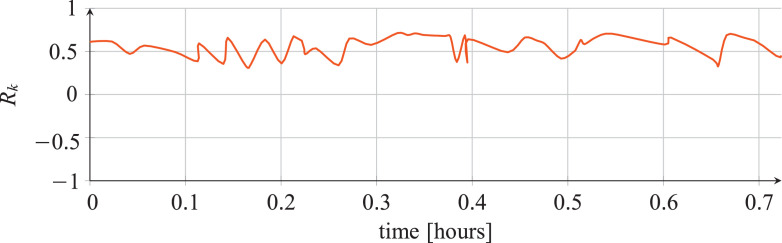
Hyper-parameters optimization plot for the tool classifier based on extra-trees.

**Fig. 6:**
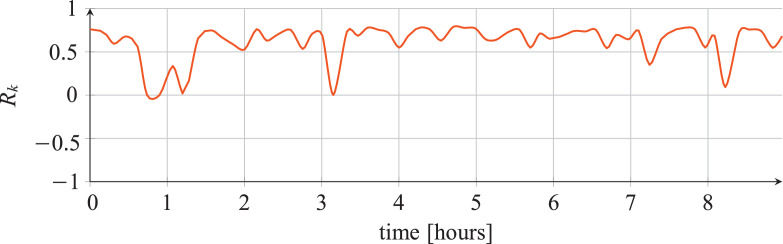
Hyper-parameters optimization plot for the tool classifier based on neural network.

**Fig. 7:**
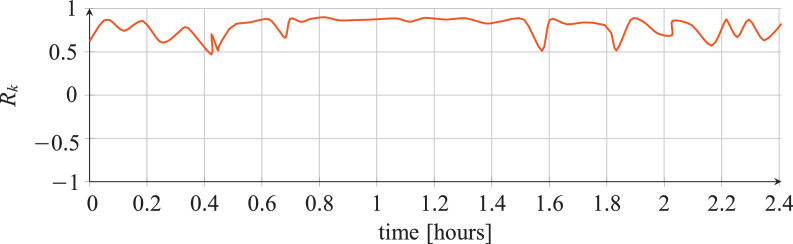
Hyper-parameters optimization plot for the tool instance classifier based on random forest.

**Fig. 8:**
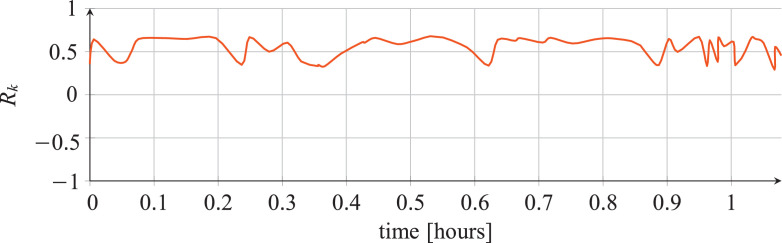
Hyper-parameters optimization plot for the tool instance classifier based on extra-trees.

**Fig. 9:**
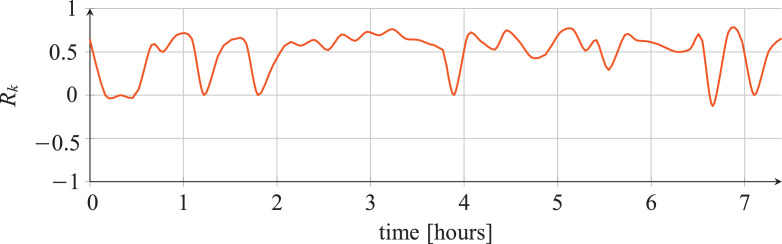
Hyper-parameters optimization plot for the tool instance classifier based on neural network.
• The tables listing the optimal hyper-parameters found by our Bayesian optimization process (Tables 5, 9, 13, 17, 21, 25, 29, 33, and 37) – we normally used the default values for the hyper-parameters not reported^1^.

**Table 5:** Optimal hyper-parameters for the category classifier based on random forest.

hyper-parameter	Value
criterion	Entropy
max_depth	17
min_samples_leaf	9
min_samples_split	38
n_estimators	89
• The tables reporting several classification statistics computed on the training set, development set, known tools test Set and unknown tools test set (Tables 6, 10, 14, 18, 22, 26, 30, 34 and 38).

**Table 6:** Classification statistics for the category classifier based on random forest.

statistic	training set	dev set	KTS	UTS
samples	955,872	119,484	119,485	30,144
accuracy [%]	97.702	97.542	97.447	40.459
balanced accuracy [%]	95.568	93.997	94.041	34.031
precision [%]	86.660	85.637	85.226	32.723
recall [%]	95.568	93.997	94.041	34.031
Cohen's kappa [%]	86.105	85.021	84.577	1.097
F-score [%]	90.719	89.459	89.230	32.151
Jaccard score [%]	83.556	81.607	81.255	20.631
Hamming loss	0.023	0.025	0.026	0.595
zero-one loss	0.023	0.025	0.026	0.595
*R_k_*	0.865	0.854	0.850	0.011
• The confusion matrices for each classifier (Tables 7, 11, 15, 19, 23, 27, 31, 35, and 39).

**Table 7:** Confusion matrix for the category classifier based on random forest on the KTS.

		inferred		
		browser	crawler	dos
target	browser	6872	194	322
	crawler	120	2112	83
	dos	1980	352	107,450
• The results of the classification of the unknown tools (Tables 8, 12, 16, 20, 24, 28, 32, 36, and 40).

**Table 8:** Classification of unknown tools for the category classifier based on random forest.

inferred class	samples	inferred class	samples
browser	3291	browser	1369
crawler	2623	crawler	883
dos	620	dos	1413
	
(a) Classification of firefox-68.0.		(b) Classification of grabsite-2.1.16.	

browser	6149	browser	6657
crawler	605	crawler	2465
dos	2196	dos	1873
	
(c) Classification of opera-62.0.3331.66.		(d) Classification of slowhttptest-1.6.

**Table 9:** Optimal hyper-parameters for the category classifier based on extra-trees.

hyper-parameter	value
criterion	entropy
max_depth	20
min_samples_leaf	3
min_samples_split	37
n_estimators	88

**Table 10:** Classification statistics for the category classifier based on extra-trees.

statistic	training set	dev set	KTS	UTS
samples	955,872	119,484	119,485	30,144
accuracy [%]	85.085	85.033	85.015	37.646
balanced accuracy [%]	80.692	80.216	80.373	38.510
precision [%]	62.558	62.265	62.413	33.339
recall [%]	80.692	80.216	80.373	38.510
Cohen's kappa [%]	42.760	42.372	42.547	1.694
F-score [%]	62.138	61.837	61.866	33.949
Jaccard score [%]	51.661	51.322	51.552	21.426
Hamming loss	0.149	0.150	0.150	0.624
zero-one loss	0.149	0.150	0.150	0.624
*R_k_*	0.491	0.487	0.489	0.017

**Table 11:** Confusion matrix for the category classifier based on extra-trees on the KTS.

		inferred		
		browser	crawler	dos
target	browser	5255	1496	667
	crawler	126	1954	235
	dos	1439	13,942	94,401

**Table 12:** Classification of unknown tools for the category classifier based on extra-trees.

inferred class	samples	inferred class	samples
browser	2570	browser	837
crawler	1242	crawler	1809
dos	2722	dos	1019
	
(a) Classification of firefox-68.0.		(b) Classification of grabsite-2.1.16.	

inferred class	samples	inferred class	samples
browser	5237	browser	5077
crawler	1046	crawler	4186
dos	2667	dos	1732
	
(c) Classification of opera-62.0.3331.66.		(d) Classification of slowhttptest-1.6.

**Table 13:** Optimal hyper-parameters for the category classifier based on neural network.

hyper-parameter	value
lr	0.0027014308955057255
module layers	4
module neurons_per_layer	434
module p	0.1292524544974843

### 1.3 Category classifiers

This section reports several statistics and plots about the models for classifying the traffic into categories (e.g., browser, crawler, and dos, a.k.a. network stress tools).

### 1.4 Tool classifiers

This section reports several statistics and plots about the models for classifying the traffic into tools (e.g., goldeneye, hulk, firefox, wget, edge, httrack, chrome, rudy, slowloris, curl, and wpull).

### 1.5 Tool instance classifiers

This section reports statistics and plots about the models for classifying the traffic into tool in- stances (e.g., goldeneye-2.1, firefox-62.0, hulk-1.0, wget-1.11.4, edge-42.17134.1.0, httrack- 3.49.2, chrome-48.0.2564.109, rudy-1.0.0, chrome-68.0.3440.84, firefox-42.0, slowloris-0.1.5, curl-7.55.1, curl-7.61.0, slowloris-0.1.4, wpull-2.0.1, and wget-1.19.5).

## 2 Experimental Design, Materials and Methods

The traffic used to generate the dataset has been captured using WireShark 2.6.4 with the tshark command-line interface. The web browsing part of the traffic dataset has been generated by manually browsing the Internet, as reported in Section 4.1 of the main paper [1].

Instead, to generate the traffic for the web crawling and DDoS categories, a set of Python 3 scripts have been written, which are available at 10.5281/zenodo.5797882. For each tool instance, these scripts automatically start the traffic capture (see Table 1), execute the tool on a set of websites (prior authorization to the owners has been asked whenever needed to execute such activities), wait for tool termination, and stop the capture. Then, the captures from background traffic not generated by the analyzed tools have been discarded. Multiple truncated versions of the filtered captures have been generated. As explained in Section 4.1 of the main paper [1], by truncating the captures, it has been possible to benchmark our models on incomplete connections, to test their suitability for a live analysis scenario. For instance, Fig. 2 reports the balanced accuracy of the random forest-based category classifier in a live analysis scenario, i.e., plotting the balanced accuracy with respect to the number of exchanged packets in the analyzed connection. Subsequently, the statistics have been computed on each of the TCP flows contained in both the full and truncated traffic captures using the *TCP Statistic and Analysis Tool (Tstat)* ,^2^ one of the most used traffic measurement tools. As reported in Table 2, the models use a subset of the statistics available in the *Core/Basic TCP Set*.^3^ Client/server IP addresses and TCP ports have not been reported for anonymization purposes. They are not needed in the training process of the machine learning models. All the datasets are available in the GitHub repository mentioned above.

Finally, a dataset has been generated to train a set of machine learning models able to classify a TCP connection based on the tool category, the specific tool, and the specific version of the tool used to generate it. Section 5.1 of the main paper [1] describes in detail how the models have been trained. This document reports the hyperparameters used to train each of the models. For instance, Table 14 reports the hyperparameters used to train the category classifier based on neural networks. The trained machine learning models have been made available as joblib objects^4^ at the previously mentioned GitHub repository.

**Table 14:** Classification statistics for the category classifier based on neural network.

statistic	training set	dev set	kts	uts
samples	955,872	119,484	119,485	30,144
accuracy [%]	96.132	96.005	96.017	41.912
balanced accuracy [%]	91.123	90.207	90.308	35.058
precision [%]	75.600	74.957	74.653	33.739
recall [%]	91.123	90.207	90.308	35.058
Cohen's kappa [%]	77.644	76.827	76.988	2.587
F-score [%]	80.923	80.166	79.902	33.950
Jaccard score [%]	70.873	69.961	69.772	21.943
Hamming loss	0.039	0.040	0.040	0.581
zero-one loss	0.039	0.040	0.040	0.581
*R_k_*	0.784	0.776	0.778	0.026

**Table 15:** Confusion matrix for the category classifier based on neural network on the KTS.

		inferred		
		browser	crawler	dos
target	browser	6423	489	476
	crawler	148	2018	149
	dos	1270	2227	106,285

**Table 16:** Classification of unknown tools for the category classifier based on neural network.

balanced accuracy [%]				100

	inferred class	samples	inferred class	samples

	browser	3366	browser	2012
	crawler	358	crawler	835
	dos	2810	dos	818
	
	(a) Classification of firefox-68.0.		(b) Classification of grabsite-2.1.16.	

	inferred class	samples	inferred class	samples
	browser	6086	browser	5201
	crawler	738	crawler	3447
dos	2126	dos	2347
	
(c) Classification of opera-62.0.3331.66.		(d) Classification of slowhttptest-1.6.

**Table 17:** Optimal hyper-parameters for the tool classifier based on random forest.

hyper-parameter	value
criterion	entropy
max_depth	20
min_samples_leaf	5
min_samples_split	22
n_estimators	417

**Table 18:** Classification statistics for the tool classifier based on random forest.

statistic	training set	dev set	KTS	UTS
samples	955,872	119,484	119,485	30,144
accuracy [%]	95.716	94.977	94.953	7.680
balanced accuracy [%]	95.590	89.399	90.366	8.858
precision [%]	80.233	76.446	76.447	2.624
recall [%]	95.590	89.399	90.366	2.531
Cohen's kappa [%]	91.752	90.300	90.285	3.298
F-score [%]	85.925	81.341	81.477	2.577
Jaccard score [%]	77.432	71.180	71.442	1.572
Hamming loss	0.043	0.050	0.050	0.923
zero-one loss	0.043	0.050	0.050	0.923
*R_k_*	0.919	0.904	0.904	0.041

**Table 19:** Confusion matrix for the tool classifier based on random forest on the KTS.

		chrome	curl	edge	firefox	goldeneye	inferredhttrack	hulk	rudy	slowloris	wget	wpull
target	chrome	1999	25	166	113	110	9	12	1	3	14	22
	curl	1	273	13	3	4	11	0	1	1	12	4
	edge	73	16	2717	45	26	9	10	0	1	10	9
	firefox	140	17	74	1588	71	41	17	3	2	16	29
	goldeneye	1053	22	159	366	74,967	150	1511	107	10	31	318
	httrack	17	6	4	16	20	1229	2	0	0	0	4
	hulk	409	4	21	49	357	15	28,464	20	0	9	93
	rudy	3	0	0	1	7	2	7	321	2	0	5
	slowloris	0	2	1	9	0	20	0	1	1263	0	3
	wget	2	4	3	3	12	0	1	1	0	449	7
	wpull	2	3	4	2	10	4	0	0	0	3	184

**Table 20:** Classification of unknown tools for the tool classifier based on random forest.

inferred class	samples	inferred class	samples
chrome	688	chrome	348
curl	6	curl	41
edge	329	edge	262
firefox	2315	firefox	639
goldeneye	405	goldeneye	1440
httrack	113	httrack	186
hulk	55	hulk	185
rudy	37	rudy	26
slowloris	2	slowloris	6
wget	53	wget	207
wpull	2531	wpull	325
	
(a) Classification of firefox-68.0.		(b) Classification of grabsite-2.1.16.	

inferred class	samples	inferred class	samples
chrome	5006	chrome	175
curl	78	curl	57
edge	288	edge	1831
firefox	1013	firefox	2334
goldeneye	2139	goldeneye	1032
httrack	182	httrack	372
hulk	34	hulk	17
rudy	15	rudy	4022
slowloris	3	slowloris	39
wget	20	wget	39
wpull	172	wpull	1077
	
(c) Classification of opera-62.0.3331.66.		(d) Classification of slowhttptest-1.6.

**Table 21:** Optimal hyper-parameters for the tool classifier based on extra-trees.

hyper-parameter	value
criterion	entropy
max_depth	20
min_samples_leaf	21
min_samples_split	49
n_estimators	96

**Table 22:** Classification statistics for the tool classifier based on extra-trees.

statistic	training set	dev set	KTS	UTS
samples	955,872	119,484	119,485	30,144
accuracy [%]	83.376	83.256	83.226	4.668
balanced accuracy [%]	69.140	66.884	66.701	5.383
precision [%]	50.983	49.885	49.685	3.105
recall [%]	69.140	66.884	66.701	1.538
Cohen's kappa [%]	70.282	70.017	70.042	2.396
F-score [%]	52.837	51.522	51.257	2.057
Jaccard score [%]	39.406	38.294	38.123	1.202
Hamming loss	0.166	0.167	0.168	0.953
zero-one loss	0.166	0.167	0.168	0.953
*R_k_*	0.713	0.710	0.711	0.030

**Table 23:** Confusion matrix for the tool classifier based on extra-trees on the KTS.

		chrome	curl	edge	firefox	goldeneye	inferredhttrack	hulk	rudy	slowloris	wget	wpull
target	chrome	1256	73	208	112	358	126	43	13	153	64	68
	curl	0	179	22	0	41	38	2	1	26	7	7
	edge	124	35	2025	30	103	140	51	10	284	11	103
	firefox	204	52	171	837	286	169	27	17	68	63	104
	goldeneye	92	237	1084	57	66,908	5511	1061	173	2759	458	354
	httrack	17	15	15	14	122	978	1	1	94	13	28
	hulk	26	6	431	38	1496	1225	25,393	15	641	20	150
	rudy	0	2	4	0	32	19	5	249	33	2	2
	slowloris	1	0	2	0	0	35	0	0	1260	0	1
	wget	4	18	19	0	40	51	4	3	68	255	20
	wpull	1	5	8	1	36	38	0	6	12	3	102

**Table 24:** Classification of unknown tools for the tool classifier based on extra-trees.

inferred class	samples	inferred class	samples
chrome	260	chrome	188
curl	350	curl	142
edge	663	edge	230
firefox	1407	firefox	187
goldeneye	495	goldeneye	1097
httrack	585	httrack	468
hulk	2249	hulk	32
rudy	8	rudy	33
slowloris	41	slowloris	132
wget	215	wget	293
wpull	261	wpull	863
	
(a) Classification of firefox-68.0.		(b) Classification of grabsite-2.1.16.	

inferred class	samples	inferred class	samples
chrome	3498	chrome	373
curl	412	edge	1458
edge	494	firefox	1120
firefox	523	goldeneye	2033
goldeneye	3009	httrack	1598
httrack	354	hulk	61
hulk	39	rudy	3516
rudy	147	slowloris	463
slowloris	156	wget	53
wget	66	wpull	320
wpull	252		
	
(c) Classification of opera-62.0.3331.66.		(d) Classification of slowhttptest-1.6.

**Table 25:** Optimal hyper-parameters for the tool classifier based on neural network.

hyper-parameter	value
lr	0.0015639059764891423
module layers	4
module neurons_per_layer	196
module p	0.34048448616373395

**Table 26:** Classification statistics for the tool classifier based on neural network.

statistic	training set	dev set	KTS	UTS
samples	955,872	119,484	119,485	30,144
accuracy [%]	87.735	87.516	87.516	7.467
balanced accuracy [%]	81.721	79.971	80.332	8.613
precision [%]	53.469	52.862	52.688	2.854
recall [%]	81.721	79.971	80.332	2.461
Cohen's kappa [%]	77.740	77.340	77.379	3.560
F-score [%]	60.582	59.641	59.479	2.643
Jaccard score [%]	48.020	47.214	47.164	1.621
Hamming loss	0.123	0.125	0.125	0.925
zero-one loss	0.123	0.125	0.125	0.925
*R_k_*	0.785	0.782	0.782	0.044

**Table 27:** Confusion matrix for the tool classifier based on neural network on the KTS.

		chrome	curl	edge	firefox	goldeneye	inferredhttrack	hulk	rudy	slowloris	wget	wpull
target	chrome	1509	50	204	300	214	42	35	4	2	75	39
	curl	1	207	14	5	37	14	2	2	0	28	13
	edge	61	16	2552	66	36	28	38	10	1	51	57
	firefox	165	19	119	1269	184	80	50	10	1	34	67
	goldeneye	1189	313	821	562	67,851	710	3873	1273	106	1176	820
	httrack	13	1	37	38	98	1087	10	1	0	7	6
	hulk	367	4	53	48	488	115	27,981	147	0	133	105
	rudy	1	0	2	1	5	2	11	306	2	9	9
	slowloris	2	0	0	19	0	15	0	3	1258	1	1
	wget	4	15	16	1	17	5	2	6	0	383	33
	wpull	1	2	5	5	12	4	3	4	0	10	166

**Table 28:** Classification of unknown tools for the tool classifier based on neural network.

inferred class	samples	inferred class	samples
chrome	339	chrome	364
curl	0	curl	13
edge	717	edge	458
firefox	2251	firefox	558
goldeneye	2612	goldeneye	670
httrack	244	httrack	521
hulk	154	hulk	290
rudy	1	rudy	133
slowloris	0	slowloris	0
wget	76	wget	243
wpull	140	wpull	415
	
(a) Classification of firefox-68.0.		(b) Classification of grabsite-2.1.16.	

inferred class	samples	inferred class	samples
chrome	4097	chrome	1831
curl	138	curl	109
edge	134	edge	704
firefox	1502	firefox	1323
goldeneye	2342	goldeneye	270
httrack	367	httrack	378
hulk	80	hulk	109
rudy	29	rudy	4144
slowloris	4	slowloris	338
wget	59	wget	640
wpull	198	wpull	1149
	
(c) Classification of opera-62.0.3331.66.		(d) Classification of slowhttptest-1.6.

**Table 29:** Optimal hyper-parameters for the tool instance classifier based on random forest.

hyper-parameter	value
criterion	entropy
max_depth	20
min_samples_leaf	6
min_samples_split	18
n_estimators	314

**Table 30:** Classification statistics for the tool instance classifier based on random forest.

statistic	training set	dev set	KTS	UTS
samples	955,872	119,484	119,485	30,144
accuracy [%]	95.565	94.642	94.601	0.000
balanced accuracy [%]	95.196	87.183	88.021	0.000
precision [%]	79.996	74.641	75.061	0.000
recall [%]	95.196	87.183	88.021	0.000
Cohen's kappa [%]	91.495	89.690	89.648	0.000
F-score [%]	85.628	79.254	79.707	0.000
Jaccard score [%]	76.831	68.057	68.871	0.000
Hamming loss	0.044	0.054	0.054	1.000
zero-one loss	0.044	0.054	0.054	1.000
*R_k_*	0.916	0.898	0.898	0.000

**Table 31:** Confusion matrix for the tool instance classifier based on random forest on the KTS (where go = goldeneye, fi = firefox, hu = hulk, wg = wget, ed = edge, ht = httrack, ch = chrome, ru = rudy, sl = slowloris, cu = curl and wp = wpull.

		inferred															
		ch-48.0	ch-68.0	cu-7.55.1	cu-7.61.0	ed-42	fi-42.0	fi-62.0	go-2.1	ht-3.49.2	hu-1.0	ru-1.0.0	sl-0.1.4	sl-0.1.5	wg-1.11.4	wg-1.19.5	wp-2.0.1
target	ch-48.0	1128	80	14	4	91	18	20	61	4	4	0	0	3	3	10	10
	ch-68.0	49	810	7	1	36	18	33	53	6	4	0	0	0	0	1	6
	cu-7.55.1	0	1	150	6	0	0	3	4	5	0	0	0	1	2	0	0
	cu-7.61.0	0	0	2	122	9	2	0	0	4	0	1	0	0	2	6	3
	ed-42	75	37	2	21	2656	32	19	35	9	10	0	0	2	4	6	8
	fi-42.0	26	22	9	2	41	646	51	34	12	5	2	0	2	4	3	10
	fi-62.0	27	45	9	1	28	46	894	37	17	6	0	0	3	0	5	11
	go-2.1	114	1191	50	2	157	74	320	74,802	130	1459	93	4	5	18	10	265
	ht-3.49.2	4	15	7	1	7	5	6	23	1224	2	0	0	1	0	0	3
	hu-1.0	35	485	11	0	21	16	53	293	12	28,435	18	0	3	6	0	53
	ru-1.0.0	3	1	0	1	0	1	0	6	3	7	321	1	0	0	0	4
	sl-0.1.4	0	0	0	0	0	0	0	0	0	0	1	607	6	0	0	0
	sl-0.1.5	0	0	4	0	1	6	1	0	13	0	1	48	608	0	0	3
	wg-1.11.4	2	1	1	2	4	3	0	8	0	1	1	0	0	259	0	1
	wg-1.19.5	1	0	0	1	0	0	0	0	0	0	0	0	0	0	196	1
	wp-2.0.1	1	5	1	5	4	4	2	6	3	1	0	0	1	3	0	176

**Table 32:** Classification of unknown tools for the tool instance classifier based on random forest.

inferred class	samples	inferred class	samples
chrome-48.0.2564.109	367	chrome-48.0.2564.109	112
chrome-68.0.3440.84	226	chrome-68.0.3440.84	168
curl-7.55.1	9	curl-7.55.1	53
edge-42.17134.1.0	356	curl-7.61.0	2
firefox-42.0	607	edge-42.17134.1.0	260
firefox-62.0	1805	firefox-42.0	224
goldeneye-2.1	377	firefox-62.0	296
httrack-3.49.2	47	goldeneye-2.1	1579
hulk-1.0	49	httrack-3.49.2	164
rudy-1.0.0	33	hulk-1.0	223
slowloris-0.1.5	52	rudy-1.0.0	25
wget-1.19.5	60	slowloris-0.1.5	11
wpull-2.0.1	2546	wget-1.11.4	84
		wget-1.19.5	23
		wpull-2.0.1	441
	
(a) Classification of firefox-68.0.		(b) Classification of grabsite-2.1.16.	

inferred class	samples	inferred class	samples
chrome-48.0.2564.109	2365	chrome-48.0.2564.109	165
chrome-68.0.3440.84	2178	chrome-68.0.3440.84	27
curl-7.55.1	103	curl-7.55.1	78
curl-7.61.0	5	curl-7.61.0	22
edge-42.17134.1.0	382	edge-42.17134.1.0	1795
firefox-42.0	275	firefox-42.0	394
firefox-62.0	714	firefox-62.0	1220
goldeneye-2.1	2431	goldeneye-2.1	1212
httrack-3.49.2	231	httrack-3.49.2	341
hulk-1.0	35	hulk-1.0	33
rudy-1.0.0	18	rudy-1.0.0	4061
slowloris-0.1.5	6	slowloris-0.1.4	1
wget-1.11.4	12	slowloris-0.1.5	56
wget-1.19.5	5	wget-1.11.4	25
wpull-2.0.1	190	wget-1.19.5	8
		wpull-2.0.1	1557
	
(c) Classification of opera-62.0.3331.66.		(d) Classification of slowhttptest-1.6.

**Table 33:** Optimal hyper-parameters for the tool instance classifier based on extra-trees.

hyper-parameter	value
criterion	gini
max_depth	20
min_samples_leaf	8
min_samples_split	18
n_estimators	417

**Table 34:** Classification statistics for the tool instance classifier based on extra-trees.

statistic	training set	dev set	KTS	UTS
samples	955,872	119,484	119,485	30,144
accuracy [%]	80.889	80.672	80.644	0.000
balanced accuracy [%]	66.162	63.546	62.533	0.000
precision [%]	44.696	42.762	42.506	0.000
recall [%]	66.162	63.546	62.533	0.000
Cohen's kappa [%]	66.786	66.362	66.387	0.000
F-score [%]	45.703	43.713	43.182	0.000
Jaccard score [%]	33.113	31.442	31.131	0.000
Hamming loss	0.191	0.193	0.194	1.000
zero-one loss	0.191	0.193	0.194	1.000
*R_k_*	0.681	0.677	0.677	0.000

**Table 35:** Confusion matrix for the tool instance classifier based on extra-trees on the KTS (where go = goldeneye, fi = firefox, hu = hulk, wg = wget, ed = edge, ht = httrack, ch = chrome, ru = rudy, sl = slowloris, cu = curl and wp = wpull.

		inferred															
		ch-48.0	ch-68.0	cu-7.55.1	cu-7.61.0	ed-42	fi-42.0	fi-62.0	go-2.1	ht-3.49.2	hu-1.0	ru-1.0.0	sl-0.1.4	sl-0.1.5	wg-1.11.4	wg-1.19.5	wp-2.0.1
target	ch-48.0	721	89	62	5	44	20	23	180	66	0	9	98	48	26	39	20
	ch-68.0	92	484	22	4	85	26	20	151	45	32	0	2	6	18	20	17
	cu-7.55.1	0	0	98	5	2	0	0	34	8	2	1	2	7	13	0	0
	cu-7.61.0	0	0	2	86	12	0	0	0	18	0	0	13	10	3	3	4
	ed-42	52	93	13	58	1950	17	11	95	133	47	2	209	126	31	23	56
	fi-42.0	45	32	31	12	80	268	95	80	72	3	7	23	42	26	20	33
	fi-62.0	70	34	32	6	46	70	498	184	65	3	5	2	20	26	39	29
	go-2.1	59	72	346	366	901	11	179	64,648	5382	767	24	2364	1052	1754	660	109
	ht-3.49.2	8	5	14	1	11	2	4	118	990	1	0	6	73	32	4	29
	hu-1.0	2	42	140	52	457	3	171	1333	1129	24,964	0	612	52	43	296	145
	ru-1.0.0	0	0	1	2	6	0	1	29	17	3	236	26	13	10	4	0
	sl-0.1.4	0	0	0	0	0	0	0	0	0	0	0	525	89	0	0	0
	sl-0.1.5	0	0	1	0	0	0	0	0	25	0	0	205	453	0	0	1
	wg-1.11.4	1	1	1	6	5	0	0	22	11	0	0	5	3	212	6	10
	wg-1.19.5	0	0	1	5	3	0	0	0	21	3	0	30	0	2	132	2
	wp-2.0.1	2	0	3	9	8	0	1	35	33	0	4	4	7	12	1	93

**Table 36:** Classification of unknown tools for the tool instance classifier based on extra-trees.

inferred class	samples	inferred class	samples
chrome-48.0.2564.109	450	chrome-48.0.2564.109	148
chrome-68.0.3440.84	14	chrome-68.0.3440.84	24
curl-7.55.1	183	curl-7.55.1	80
curl-7.61.0	58	curl-7.61.0	42
edge-42.17134.1.0	573	edge-42.17134.1.0	217
firefox-42.0	342	firefox-42.0	46
firefox-62.0	1026	firefox-62.0	156
goldeneye-2.1	472	goldeneye-2.1	1039
httrack-3.49.2	596	httrack-3.49.2	404
hulk-1.0	2241	hulk-1.0	22
rudy-1.0.0	22	rudy-1.0.0	20
slowloris-0.1.4	2	slowloris-0.1.4	97
slowloris-0.1.5	23	slowloris-0.1.5	123
wget-1.11.4	49	wget-1.11.4	198
wget-1.19.5	260	wget-1.19.5	170
wpull-2.0.1	223	wpull-2.0.1	879
	
(a) Classification of firefox-68.0.		(b) Classification of grabsite-2.1.16.	

inferred class	samples	inferred class	samples
chrome-48.0.2564.109	1913	chrome-48.0.2564.109	271
chrome-68.0.3440.84	1672	chrome-68.0.3440.84	214
curl-7.55.1	503	curl-7.55.1	6
curl-7.61.0	22	curl-7.61.0	19
edge-42.17134.1.0	432	edge-42.17134.1.0	1473
firefox-42.0	192	firefox-42.0	211
firefox-62.0	220	firefox-62.0	581
goldeneye-2.1	2978	goldeneye-2.1	1965
httrack-3.49.2	341	httrack-3.49.2	920
hulk-1.0	41	hulk-1.0	32
rudy-1.0.0	166	rudy-1.0.0	2674
slowloris-0.1.4	19	slowloris-0.1.4	160
slowloris-0.1.5	80	slowloris-0.1.5	1592
wget-1.11.4	92	wget-1.11.4	226
wget-1.19.5	81	wget-1.19.5	13
wpull-2.0.1	198	wpull-2.0.1	638
	
(c) Classification of opera-62.0.3331.66.		(d) Classification of slowhttptest-1.6.

**Table 37:** Optimal hyper-parameters for the tool instance classifier based on neural network.

hyper-parameter	value
lr	0.001044660236833224
module layers	4
module neurons_per_layer	478
module p	0.33926635120188525

**Table 38:** Classification statistics for the tool instance classifier based on neural network.

statistic	training set	dev set	KTS	UTS
samples	955,872	119,484	119,485	30,144
accuracy [%]	83.050	82.687	82.649	0.000
balanced accuracy [%]	79.137	76.554	76.767	0.000
precision [%]	45.881	44.808	44.725	0.000
recall [%]	79.137	76.554	76.767	0.000
Cohen's kappa [%]	70.892	70.285	70.290	0.000
F-score [%]	50.890	49.582	49.411	0.000
Jaccard score [%]	38.186	37.084	36.897	0.000
Hamming loss	0.170	0.173	0.174	1.000
zero-one loss	0.170	0.173	0.174	1.000
*R_k_*	0.724	0.719	0.719	0.000

**Table 39:** Confusion matrix for the tool instance classifier based on neural network on the KTS (where go = goldeneye, fi = firefox, hu = hulk, wg = wget, ed = edge, ht = httrack, ch = chrome, ru = rudy, sl = slowloris, cu = curl and wp = wpull.

		inferred															
		ch-48.0	ch-68.0	cu-7.55.1	cu-7.61.0	ed-42	fi-42.0	fi-62.0	go-2.1	ht-3.49.2	hu-1.0	ru-1.0.0	sl-0.1.4	sl-0.1.5	wg-1.11.4	wg-1.19.5	wp-2.0.1
target	ch-48.0	832	178	26	14	91	57	78	43	16	6	22	0	2	8	41	36
	ch-68.0	60	716	25	1	40	23	80	45	7	8	1	0	2	3	1	12
	cu-7.55.1	0	2	133	5	0	1	1	6	11	0	0	0	1	6	0	6
	cu-7.61.0	0	0	0	121	3	1	0	1	6	0	2	0	0	3	12	2
	ed-42	38	77	2	117	2395	30	40	19	39	33	10	0	1	14	62	39
	fi-42.0	25	55	5	16	47	437	155	19	56	5	6	0	4	4	7	28
	fi-62.0	18	98	36	2	28	68	752	39	35	6	2	0	6	5	11	23
	go-2.1	132	1922	2542	50	598	123	1033	63,582	1334	2713	1230	5	45	993	719	1673
	ht-3.49.2	5	17	22	1	13	7	24	37	1150	1	1	0	4	5	3	8
	hu-1.0	2	573	76	15	60	20	474	264	123	26,812	287	0	2	23	431	279
	ru-1.0.0	2	0	1	0	1	1	0	1	5	9	312	2	0	7	0	7
	sl-0.1.4	0	0	0	0	0	0	0	0	0	0	2	538	74	0	0	0
	sl-0.1.5	0	0	0	1	0	5	0	0	27	0	1	248	402	0	1	0
	wg-1.11.4	0	2	2	7	2	1	1	13	8	0	5	0	0	228	2	12
	wg-1.19.5	0	0	0	6	4	0	0	1	0	0	0	0	0	0	185	3
	wp-2.0.1	1	8	2	6	5	3	0	3	9	0	5	0	1	8	3	158

**Table 40:** Classification of unknown tools for the tool instance classifier based on neural network.

inferred class	samples	inferred class	samples
chrome-48.0.2564.109	142	chrome-48.0.2564.109	210
chrome-68.0.3440.84	336	chrome-68.0.3440.84	321
curl-7.55.1	62	curl-7.55.1	47
curl-7.61.0	3	curl-7.61.0	19
edge-42.17134.1.0	454	edge-42.17134.1.0	673
firefox-42.0	660	firefox-42.0	139
firefox-62.0	1651	firefox-62.0	439
goldeneye-2.1	2386	goldeneye-2.1	480
httrack-3.49.2	351	httrack-3.49.2	312
hulk-1.0	62	hulk-1.0	121
rudy-1.0.0	60	rudy-1.0.0	240
slowloris-0.1.4	0	slowloris-0.1.4	0
slowloris-0.1.5	75	slowloris-0.1.5	9
wget-1.11.4	5	wget-1.11.4	99
wget-1.19.5	96	wget-1.19.5	201
wpull-2.0.1	191	wpull-2.0.1	355
	
(a) Classification of firefox-68.0.		(b) Classification of grabsite-2.1.16.	

inferred class	samples	inferred class	samples
chrome-48.0.2564.109	1636	chrome-48.0.2564.109	920
chrome-68.0.3440.84	3446	chrome-68.0.3440.84	239
curl-7.55.1	509	curl-7.55.1	33
curl-7.61.0	8	curl-7.61.0	71
edge-42.17134.1.0	75	edge-42.17134.1.0	1022
firefox-42.0	281	firefox-42.0	473
firefox-62.0	1091	firefox-62.0	1262
goldeneye-2.1	1292	goldeneye-2.1	100
httrack-3.49.2	322	httrack-3.49.2	735
hulk-1.0	26	hulk-1.0	61
rudy-1.0.0	20	rudy-1.0.0	4359
slowloris-0.1.4	0	slowloris-0.1.4	9
slowloris-0.1.5	7	slowloris-0.1.5	3
wget-1.11.4	45	wget-1.11.4	405
wget-1.19.5	28	wget-1.19.5	129
wpull-2.0.1	164	wpull-2.0.1	1174
	
(c) Classification of opera-62.0.3331.66.		(d) Classification of slowhttptest-1.6.	

## CRediT authorship contribution statement

**Daniele Canavese:** Conceptualization, Methodology, Software, Validation, Writing – original draft. **Leonardo Regano:** Conceptualization, Validation, Writing – original draft. **Cataldo Basile:** Conceptualization, Investigation, Writing – review & editing. **Gabriele Ciravegna:** Software, Writing – original draft. **Antonio Lioy:** Resources, Writing – review & editing.

## Declaration of Competing Interest

The authors declare that they have no known competing financial interests or personal relationships which have, or could be perceived to have, influenced the work reported in this article.

## Footnotes

2 http://tstat.polito.it/

3 http://tstat.polito.it/measure.shtml#log_tcp_complete

4 https://scikit-learn.org/stable/modules/model_persistence.html

## Footnotes

1 The most notable exceptions are given by the neural networks’ batch size and number of epochs, that we chose to set to 1024 and 50, respectively.

## References

[1] Canavese D., Regano L., Basile C., Ciravegna G., Lioy A. (2021). Encryption agnostic classifiers of traffic originators and their application to anomaly detection. Comput. Electr. Eng..

